# Supplementary material for: The influence of rhizosphere soil fungal diversity and complex community structure on wheat root rot disease
Source: PeerJ. 2021 Dec 13;9:e12601. doi: 10.7717/peerj.12601 (PMC8675258; doi:10.7717/peerj.12601)
Supplement: Supplemental Information 1 — After the occurrence of wheat root rot disease, the ears of wheat is be coming abnormal white, and even false ripening in advance. [file peerj-09-12601-s001.docx]

Supplementary picture information on wheat root rot symptoms


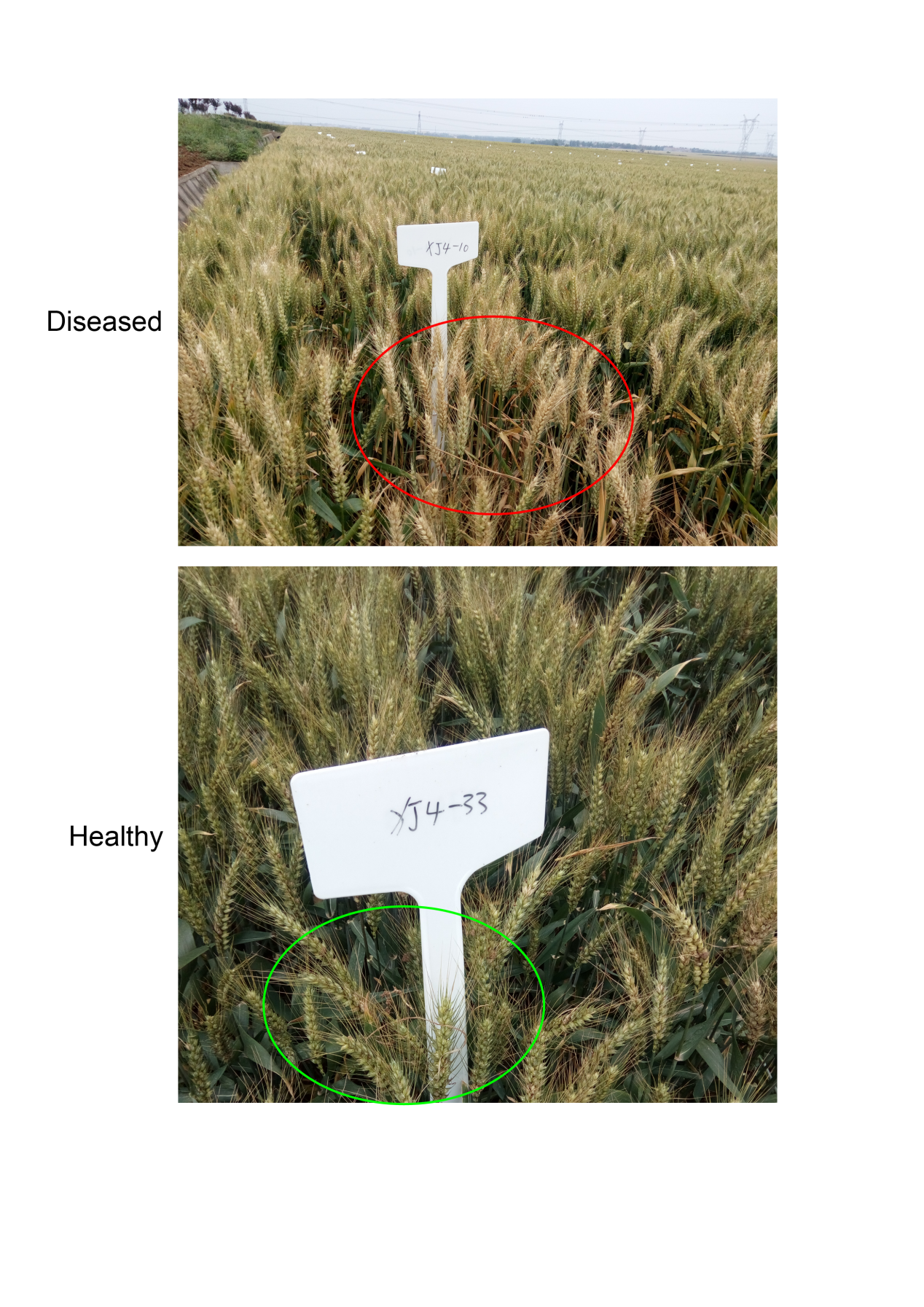


***This picture mainly shows the readers the serious situation of the root rot disease in the wheat field of Xiangyang Original Farm. After the occurrence of wheat root rot disease, the ears of wheat is becoming abnormal white, and even false ripening in advance.  Judging by this phenotype, when the roots of wheat plants were dug out, it was observed that the diseased roots were unusually brown, dark brown, and even black compared to the healthy roots.  These diseased roots can lead to plant death at a later stage, leading to crop failure.***
