# Supplementary material for: The influence of rhizosphere soil fungal diversity and complex community structure on wheat root rot disease
Source: PeerJ. 2021 Dec 13;9:e12601. doi: 10.7717/peerj.12601 (PMC8675258; doi:10.7717/peerj.12601)
Supplement: Supplemental Information 2 [file peerj-09-12601-s002.docx]

| Sample ID | Total phosphorus (TP,g/kg) | Ammonium nitrogen (NH4,mg/kg) | Nitrate nitrogen (NO3,mg/kg) | Total nitrogen (TN,mg/kg) | pH | Soil density (SD,g/cm3) | Total carbon (TC,mg/g) | Soil dry-humidity ratio (DHR) |
| --- | --- | --- | --- | --- | --- | --- | --- | --- |
| H4_1 | 4.3763 | 21.2863 | 21.6210 | 235.0840 | 7.1700 | 2.7200 | 8.9200 | 84.7787 |
| H4-2 | 4.1800 | 16.7560 | 32.0440 | 164.1807 | 7.1600 | 2.6300 | 9.7400 | 85.6045 |
| H4_3 | 4.0789 | 13.2727 | 26.7060 | 234.0336 | 7.2700 | 2.7000 | 11.4700 | 88.2627 |
| H4-4 | 4.1275 | 13.5000 | 32.4540 | 184.6639 | 7.1700 | 2.7522 | 8.1300 | 86.7650 |
| H4_5 | 4.0500 | 15.3000 | 19.0650 | 202.5210 | 7.1200 | 2.6100 | 10.8500 | 85.9040 |
| D4_1 | 4.2185 | 17.1800 | 23.5610 | 240.3361 | 7.0700 | 2.4179 | 8.6300 | 85.1814 |
| D4-2 | 4.0728 | 15.3500 | 26.6460 | 246.6387 | 6.8600 | 2.3500 | 8.5800 | 86.2243 |
| D4_3 | 3.9272 | 14.8800 | 37.6470 | 279.2017 | 6.9900 | 2.4800 | 10.1200 | 84.9437 |
| D4-4 | 4.4431 | 12.0909 | 31.1620 | 265.5400 | 6.9300 | 2.4000 | 11.5100 | 85.4740 |
| D4_5 | 3.9697 | 10.6818 | 35.8160 | 225.6900 | 6.8900 | 2.4500 | 13.9600 | 83.9044 |
| H5_1 | 3.8058 | 21.3200 | 22.7070 | 189.9160 | 7.0800 | 2.5283 | 8.4800 | 89.5503 |
| H5-2 | 3.5508 | 19.4545 | 26.5860 | 289.7059 | 7.1300 | 2.4991 | 7.6100 | 89.3333 |
| H5_3 | 3.9697 | 10.4091 | 21.4990 | 160.5042 | 7.2600 | 2.5048 | 7.0100 | 89.8389 |
| H5-4 | 3.7800 | 18.8700 | 24.9940 | 208.8235 | 7.1200 | 2.5060 | 8.7900 | 89.9771 |
| H5_5 | 4.0000 | 17.2187 | 21.6360 | 239.2857 | 7.1600 | 2.4585 | 7.7600 | 90.1297 |
| D5_1 | 3.6176 | 18.3800 | 31.5000 | 260.2941 | 6.9400 | 2.4780 | 7.9400 | 89.0790 |
| D5-2 | 3.8300 | 16.5300 | 34.6890 | 152.1008 | 6.8600 | 2.5315 | 8.6300 | 88.7516 |
| D5_3 | 3.7876 | 21.4091 | 28.9700 | 240.3361 | 6.7800 | 2.4700 | 9.0400 | 88.5697 |
| D5-4 | 3.9200 | 16.2565 | 26.5800 | 294.9580 | 6.6100 | 2.4557 | 8.3200 | 88.8673 |
| D5_5 | 4.0425 | 20.9545 | 25.1660 | 239.2857 | 6.6100 | 2.4452 | 8.7800 | 88.5789 |

Table 3

*H,health; H4,samples from April; H5,samples from May; D,disease, D4,samples from April; D5,samples from May
